# Single nucleotide polymorphisms of *GYS2* gene and its association with milk production traits of dairy cows

**DOI:** 10.1080/10495398.2024.2432966

**Published:** 2024-12-04

**Authors:** Wen Ye, Ao Chen, Lingna Xu, Dongxiao Sun, Bo Han

**Affiliations:** a Department of Animal Genetics and Breeding, College of Animal Science and Technology, National Engineering Laboratory for Animal Breeding, Key Laboratory of Animal Genetics, Breeding and Reproduction of Ministry of Agriculture and Rural Affairs, State Key Laboratory of Animal Biotech Breeding, China Agricultural University, Beijing, China; b Beijing Jingwa Agricultural Innovation Center, Beijing, China

**Keywords:** *GYS2*, SNP, genetic effect, milk production traits, dairy cattle

## Abstract

Glycogen synthase 2 (*GYS2*) encodes liver glycogen synthase, a rate limiting enzyme in glycogen metabolism. Our preliminary work suggested that *GYS2* was a candidate gene affecting milk production traits by analyzing the liver proteome of dairy cows. Herein, this research identified single nucleotide polymorphisms (SNPs) of *GYS2*, analyzed their genetic effects on traits of dairy cattle, and speculated the pathogenic mechanism through functional prediction of key mutation sites. Seven SNPs were found by resequencing and the association analysis showed that these SNPs were significantly associated with 305-day milk yield, fat yield, protein yield or fat percentage (*p*-value ≤ 0.0488). Six SNPs among them formed two haplotype blocks and they were associated with 305-day milk yield, fat yield, protein yield or fat percentage (*p*-value ≤ 0.0349). Furthermore, 5:g.88602007G > A and 5:g.88602026G > A were predicted to change the transcription factor binding sites (TFBSs), which might regulate the expression of *GYS2*. The missense mutation site, 5:g.88602535G > T, changed the secondary structure of mRNA and the secondary and tertiary structure of protein. In summary, the *GYS2* was proved to have genetic effect on milk production traits, and its valuable seven SNPs, could provide more useful genetic information for molecular breeding of dairy cows.

## Graphical Abstract

**Figure:**
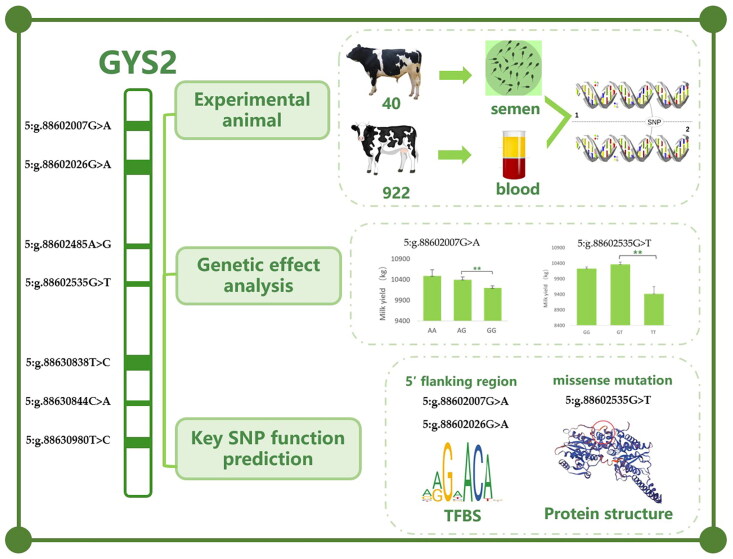

## Introduction

With the improvement of economic level and the awareness of healthy diet, the consumption of milk is surging. Meanwhile, indicators that signify milk production traits such as 305-day milk yield, fat yield, fat percentage, protein yield and protein percentage are increasingly receiving attention. Among them, high yield can increase economic value, and changes in percentage can affect milk quality. To improve milk production traits controlled by multiple genes with micro effects, individuals with specific genotypes of related genes can be screened through molecular breeding techniques. Since 2009, the genomic selection (GS) have been successively applied to dairy cattle’s breeding in many countries, which selected target traits through single nucleotide polymorphism (SNP) markers to improve selection efficiency and accuracy.^1^ As SNPs in candidate functional genes with significant genetic effects can affect milk production traits, adding relevant information to expand SNP marker data can improve the accuracy of predicting genomic estimated breeding value.^2^ However, as the available SNP markers from SNP markers pools or public databases without complete information or clear functions are numerous, it’s necessary to select and mine critical ones contributing to high breeding values accurately.^3^ So far, as the recognized major genes affecting milk production traits in cows are only *DGAT1* and *GHR*, though many functional genes and their key SNPs have been identified, we still need to explore more effective functional genes/loci.^4–6^

We previously analyzed the proteomes of liver tissues of Chinese Holstein cows at different lactation stages, and found that *GYS2* participated in the milk synthesis process of dairy cows.^7^ *GYS2* encodes hepatic glycogen synthase, a rate-limiting enzyme in the process of glycogen metabolism.^8^ It is mainly involved in pathways including AMPK, glucagon and adipocytokine signaling, which are significantly differentially expressed in liver tissues during lactations.^9^ At the onset of lactation in dairy cows, *GYS2* is activated by stimulations of the mechanism involving the liver-specific glycogen-targeted subunit GL, resulting in drastically decreased liver glycogen concentration and elevated intracellular glucose level, contributing to more energy for breast epithelial cells.^10^ In addition, multiple studies reported the presence of QTLs on BTA5 (76 to 107 Mb) related to milk production traits.^11–14^ *GYS2* (chr.5: 88602173-88658602) is located closely to the peak of several QTL regions for milk fat percentage in dairy cows.^15^ Therefore, from the perspective of both biological function and physical location, *GYS2* may be in a linked state with QTLs related to milk production traits. Khatib et al.^16^ mapped the gene *OPN* on bovine chromosome 6 based on a QTL that affected milk production traits. After conducting association analysis, they found that it had significant additive effects on fat percentage, protein percentage, and fat yield. However, there is currently few studies about the association between *GYS2* and milk production traits, so it is necessary to conduct research and identify SNPs in *GYS2* associated with milk production traits.

The objective of this study was to analyze the genetic effects of the SNPs of candidate gene *GYS2*, on 305-day milk yield, fat yield, fat percentage, protein yield, and protein percentage, and then to provide some reference information for their application on GS chip development in dairy cattle. In addition, we conducted functional predictions of key mutation sites, laying a research foundation for further exploring the causal mutations related to important traits in dairy cows.

## Materials and methods

### Experimental animal, pedigree, and phenotypic data

A total of 922 daughters of 40 Chinese Holstein bull families (no kinship) selected as the experimental population, from 22 farms belong to Beijing Sunlon Livestock Development Co., Ltd. (Beijing, China). They were raised between 2009 and 2015, with good physical health, standardized Dairy Herd Improvement (DHI) records, and genealogical information for three generations. Among them, the average number of daughters per bull mentioned above was 23 (ranging from 6 to 65). The indicators gained from DHI included 305-day milk yield (total milk volume produced over 305 days), fat yield (quantity of fat extracted from the milk), fat percentage (ratio of fat to the total milk volume), protein yield (quantity of protein extracted from the milk), and protein percentage (ratio of protein to the total milk volume) in the first and second lactations (Table S1), and the measurement method of DHI referred to the national standard of ‘Technical Specification of Chinese Holstein cattle performance test’ (standard number NY/T 1450-2007). The study was conducted in accordance with Guide for the Care and Use of Laboratory Animals and approved by the Institutional Animal Care and Use Committee (IACUC) at China Agricultural University (Beijing, China; permit number: DK996).

### Genomic DNA extraction

Genomic DNA was extracted from blood samples using TIANamp Blood DNA Kits (Tiangen Biochemical Technology, Beijing, China) and from semen samples by the optimized high-salt method. NanoDrop2000 spectrophotometer (Thermo Science, Hudson, NH, USA) and gel electrophoresis (1%; Fig. S1) were used to test the concentration and integrity of DNA, respectively. It was ensured that the values of OD_260_/OD_280_ were above 1.8, OD_260_/OD_230_ were between 1.80-2.00 and the DNA concentration was above 300 ng/μL (frozen sperm) and 100 ng/μL (blood sample) respectively to meet the requirements of subsequent experiments.

### Polymorphism detection of GYS2 gene

Primer 3.0 (https://bioinfo.ut.ee/primer3-0.4.0/) was used to design primers (Table S2) covering the sequence of *GYS2* gene’s whole coding region, and 2200 bp of upstream and downstream regulatory regions of the cattle in GenBank (Accession Number: NC_037332) (https://www.ncbi.nlm.nih.gov/nuccore). 1 μl each of diluted DNA (50 ng/μ L) was taken from semen into a mixing pool for PCR amplification (Table S2). After the amplification, products were detected by 2% gel electrophoresis, and the qualified products were sent to Beijing Qingke Xinye Biotechnology Co., Ltd (Beijing, China) for bidirectional sequencing. Chromas 1.62 software was used to view the sequencing map and obtain the sequencing sequence, and NCBI-blast (https://blast.ncbi.nlm.nih.gov/Blast.cgi) was utilized to conduct sequence alignment with the reference sequence (ARS-UCD1.2) so as to find SNP sites. Subsequently, based on the identified SNPs, Genotyping by Target Sequencing (GBTS) technology was used for genotyping the blood samples of 922 cows, which was completed by Boruidi Biotechnology Co., Ltd. (Shijiazhuang, Hebei, China). Gene frequencies and genotype frequencies of each SNP locus were calculated, and Hardy–Weinberg Equilibrium through chi square test were verified to determine whether the locus is genetically balanced in the target population.

### Linkage disequilibrium (LD) estimation

The extent of LD between the identified SNPs was estimated using Haploview 4.2 (Broad Institute of MIT and Harvard, Cambridge, MA, USA). The extent of LD is measured by the D′ value, which is proportional to it. The haplotype block with a frequency greater than 0.05 was retained.

### Association analysis between single marker/haplotype and milk production traits

The MIXED process in SAS 9.4 software (SAS Institute Inc., Cary, NC, USA) was used to conduct association analysis between SNPs or haplotype blocks and five milk production traits, and the animal model used was as follows:

y =μ+ HYS + b × M + G + a + e

where Y is the phenotype values of individual milk production traits (305-day milk yield, fat yield, fat percentage, protein yield, and protein percentage); μ is the overall mean; HYS is the fixed effect of farm, calving year, and calving season; M is the age of calving as a covariant; b is the regression coefficient of the covariate M; G is the genotype or haplotype combination effect; a is the individual random additive genetic effect, where the distribution is $\text{N }(0,A\delta_{a}^{2})$, A is a pedigree-based relationship matrix, and the additive genetic variance is $\delta_{a}^{2}$; and e is the random residual, where the distribution is $\text{N }\left(0,I\delta_{e}^{2}\right)$, the unit matrix is I, and the residual ­variance is $\delta_{e}^{2}$.

To add, $\text{Var}\left(a\right)=G=A\delta_{a}^{2}$. In animal models, A matrix is an additive genetic correlation matrix between all animal individuals, and each element of A matrix can be calculated using the following recursive formula:

$$\alpha_{\text{ii}}=\left\{ \begin{array}{l} 1+0.5\alpha_{\text{sidi}}\;\text{when}\;\text{both}\;s_{i}\;\text{and}\;d_{i}\;\text{are}\;\text{known}\;\\ 1\;\text{when}\;\text{both}\;s_{i}\;\text{and}\;d_{i}\;\text{are}\;\text{unknown} \end{array}\right.$$

$$\alpha_{\text{ij}}=\alpha_{\text{ji}}=\left\{ \begin{array}{l} 0.5\left(\alpha_{\text{isj}}+\alpha_{\text{sidj}}\right)\text{when}\;\text{both}\;s_{i}\;\text{and}\;d_{i}\;\text{are}\;\text{known}\\ 0.5\alpha_{\text{isj}}\;\text{when}\;s_{i}\;\text{is}\;\text{known}\;\text{and}\;d_{i}\;\text{is}\;\text{unknown}\\ 0.5\alpha_{\text{idj}}\text{ when }s_{i}\text{ is unknown and }d_{i}\text{ is known}\\ 0\text{ when both }s_{i}\text{ and }d_{i}\text{ are unknown } \end{array}\right.$$

Among them, $s_{i}(s_{j})$ and $d_{i}(d_{j})$ are the sire and dam of individual i (j).

In addition, the additive, dominant, and substitution effects of SNP loci were calculated, using the following formula:

a = (AA − BB) /2, d = AB − (AA + BB)/2, α = a + (q −p) × d

where, a, d, and α are the additive effect, dominant effect, and substitution effect, respectively; AA, AB, and BB are the phenotypic least squares means of the corresponding genotypes; p is the frequency of allele A; and q is the frequency of allele B.

### Biological function prediction

#### Prediction of transcription factor binding sites

Jaspar (http://jaspar.genereg.net/) and Meme-suite (http://meme-suite.org/) software were used to predict whether SNPs in the 5′ flanking region *GYS2* gene changed the TFBS. As the two sorts of software use different data sources and prediction methods, the same transcription factors obtained were chosen after screening with a relative score greater than 0.8 and the binding site located in a highly conserved core sequence (*P*-value ≤ 0.01), in order to improve the reliability of the prediction.

### Prediction of protein structure and function

RNAfold web server online prediction software (http://rna.tbi.univie.ac.at/cgi-bin/RNAWebSuite/RNAfold.cgi) was used to predict the change in the secondary structure and minimum free energy (MFE) of mRNA caused by the missense mutation identified in *GYS2* gene. Prabi software (https://npsa-prabi.ibcp.fr/cgi-bin/npsa_automat.pl?page=npsa_sopma.html), SWISS MODEL software (https://swissmodel.expasy.org/) and Mu-Pro software (http://mupro.proteomics.ics.uci.edu/) were used to predict the secondary, tertiary structure and stability change of protein on account of the changed amino acid sequence caused by the missense mutation, respectively. PolyPhen-2 software (http://genetics.bwh.harvard.edu/pph2/) was used to predict whether protein sequence function changed due to mutation, and the prediction results were Probably damaging (score ≥ 0.909), Possibly damaging (0.447 ≤ score ≤ 0.909) or Benign (harmless, score ≤ 0.446). SMART software (http://smart.embl-heidelberg.de/) was used to predict protein functional domain in coding regions.

## Results

### SNPs identification

Seven SNPs in *GYS2* in this study were found totally (Table 1), 5:g.88602007G > A (rs211260706) and 5:g.88602026G > A (rs109757669) were located in the 5′ flanking region, 5:g.88602485A > G (rs379411484) in the 5′ untranslated region (UTR), 5:g.88602535G > T (rs382704394), 5:g.88630838T > C (rs109017739), and 5:g.88630844C > A (rs111005202) in exon, and 5:g.88630980T > C (rs109503950) in intron. Two SNPs, 5:g.88630838T > C and 5:g.88630844C > A, were identified as synonymous mutations, and 5:g.88602535G > T was identified as an exonic missense, whose mutation led the 17th codon of transcribed mRNA to alter from CGG to CUG, and thus contributed to the 17th amino acid in the translated amino acid sequence change from arginine (R) to leucine (L). The genotypic and allelic frequencies of all the identified SNPs were summarized in Table 1. The Hardy Weinberg test indicated that these SNP sites reached genetic equilibrium in the experimental population.

**Table 1.** Frequencies of genotypes and alleles of identified SNPs in glycogen synthase 2 (*GYS2*) gene.

SNP Name	RS ID	Position (ARS-UCD1.2)	Gene Region	Genotype	Genotypic Frequency	Allele	Allelic Frequency	Hardy–Weinberg Equilibrium
5:g.88602007G > A	rs211260706	Chr5:88602007	5′ flanking region	AA	0.0282	A	0.1518	T
				AG	0.2473	G	0.8482	
				GG	0.7245			
5:g.88602026G > A	rs109757669	Chr5:88602026	5′ flanking region	AA	0.1909	A	0.4458	T
				AG	0.5098	G	0.5542	
				GG	0.2993			
5:g.88602485A > G	rs379411484	Chr5:88602485	5′ untranslated region (UTR)	AA	0.8709	A	0.9349	T
				AG	0.1280	G	0.0651	
				GG	0.0011			
5:g.88602535G > T	rs382704394	Chr5:88602535	exon (missense)	GG	0.7538	G	0.8709	T
				GT	0.2343	T	0.1291	
				TT	0.0119			
5:g.88630838T > C	rs109017739	Chr5:88630838	exon (synonymous)	CC	0.0824	C	0.2918	T
				CT	0.4187	T	0.7082	
				TT	0.4989			
5:g.88630844C > A	rs111005202	Chr5:88630844	exon (synonymous)	AA	0.0824	A	0.2918	T
				AC	0.4187	C	0.7082	
				CC	0.4989			
5:g.88630980T > C	rs109503950	Chr5:88630980	intron	CC	0.0813	C	0.2901	T
				CT	0.4176	T	0.7099	
				TT	0.5011			

Note: T indicates that the gene and genotype frequencies conform to Hardy–Weinberg equilibrium law, while F indicates that they do not.

### SNP association analysis

The associations between the seven SNPs of the *GYS2* gene and five milk production traits in the first and second lactation stages, including 305-day milk yield, fat yield, fat percentage, protein yield, and protein percentage, were analyzed. The results showed that the seven SNPs of *GYS2* were all significantly associated with the milk yield traits, 305-day milk yield, fat yield, and/or protein yield in the first and second lactations (*p*-value ≤ 0.0488; Table 2). Seven SNPs all had significant association with the 305-day milk yield in the first lactation (*p*-value ≤ 0.006). Five SNPs, 5:g.88602007G > A, 5:g.88602026G > A, 5:g.88630838T > C, 5:g.88630844C > A and 5:g.88630980T > C had significant association with fat yield in the first lactation (*p*-value ≤ 0.0057). Six SNPs, 5:g.88602007G > A, 5:g.88602485A > G, 5:g.88602535G > T, 5:g.88630838T > C, 5:g.88630844C > A and 5:g.88630980T > C reached significant association levels of protein yield in the first lactation (*p-*value ≤ 0.0339). In the second lactation, five SNPs, 5:g.88602485A > G, 5:g.88602535G > T, 5:g.88630838T > C, 5:g.88630844C > A and 5:g.88630980T > C reached significant association levels of the milk yield traits (*p-*value ≤ 0.0129). Additionally, there were two SNPs in the 5′ flanking region, 5:g.88602007G > A (*p*-value = 0.0054) and 5:g.88602026G > A (*p*-value = 0.008), having significant association with the fat percentage in the second lactation. The results of additive, dominant, and substitution effects were shown in Table S3.

**Table 2.** Associations of seven SNPs in *GYS2* gene with milk yield and composition traits in Chinese Holstein cattle during first and second lactations.

SNPs	Lactation	Genotype (No.)	Milk Yield (kg)	Fat Yield (kg)	Fat Percentage (%)	Protein Yield (kg)	Protein Percentage (%)
5:g.88602007G > A	1	AA (26)	10483^AB^±157.47	339.19^AB^±6.47	3.25 ± 0.06	308.17^AB^±4.72	2.95 ± 0.04
		AG (228)	10394 ^A^±74.39	344.88^A^±3.24	3.34 ± 0.03	308.07^A^±2.36	2.98 ± 0.02
		GG (668)	10192^B^±60.49	337.10^B^±2.72	3.34 ± 0.03	302.55^B^±1.99	2.98 ± 0.02
		*p*-value	0.0012**	0.0048**	0.3135	0.0051**	0.7334
	2	AA (18)	11100 ± 192.33	380.50 ± 7.89	3.37^A^±0.08	328.93 ± 5.75	2.97 ± 0.05
		AG (163)	10743 ± 82.32	381.10 ± 3.57	3.55^AB^±0.03	317.80 ± 2.60	2.97 ± 0.02
		GG (447)	10695 ± 64.21	382.42 ± 2.89	3.60^B^±0.03	316.00 ± 2.10	2.96 ± 0.02
		*p*-value	0.0949	0.8925	0.0054**	0.0613	0.9039
5:g.88602026G > A	1	AA (176)	10309^ABa^±79.28	347.49^A^±3.42	3.38 ± 1.91	307.66 ± 2.85	2.98 ± 1.91
		AG (470)	10291^Aa^±63.08	338.46^B^±2.81	3.31 ± 1.32	305.22 ± 2.28	2.97 ± 1.32
		GG (276)	10113^Bb^±71.37	334.44^B^±3.14	3.34 ± 1.55	302.19 ± 2.57	2.99 ± 1.55
		*p*-value	0.006**	0.0001**	0.9992	0.1237	0.9999
	2	AA (126)	10731 ± 87.98	377.20^Aa^±3.77	3.52^A^±0.04	316.26 ± 2.75	2.96 ± 0.02
		AG (326)	10704 ± 67.02	380.96^ABa^±2.98	3.58^AB^±0.03	316.06 ± 2.17	2.96 ± 0.02
		GG (176)	10729 ± 82.53	389.20^Bb^±3.60	3.64^A^±0.03	318.65 ± 2.62	2.98 ± 0.02
		*p*-value	0.9167	0.0048**	0.008**	0.4942	0.7088
5:g.88602485A > G	1	AA (803)	10271^a^±59.70	339.49 ± 2.70	3.33 ± 1.03	305.51 ± 2.16	2.98 ± 1.12
		AG (118)	10051^b^±92.48	335.03 ± 3.94	3.35 ± 2.21	299.9 ± 3.32	2.99 ± 2.23
		GG (1)	8899^ab^±729.24	289.06 ± 29.44	3.34 ± 21.25	252.1 ± 25.98	2.87 ± 21.25
		*p*-value	0.0058**	0.1073	0.9999	0.0236*	1
	2	AA (542)	10731 ^A^±62.84	383.15^Aa^±2.84	3.59 ± 0.03	317.39^ABa^±2.06	2.97 ± 0.02
		AG (85)	10576 ^A^±104.10	373.5^Ac^±4.41	3.55 ± 0.04	311.16^Aa^±3.22	2.95 ± 0.03
		GG (1)	13181^B^±817.35	490.73^Bb^±33.18	3.75 ± 0.33	387.57^Bb^±24.21	2.89 ± 0.19
		*p*-value	0.0028**	0.0002**	0.5172	0.0012**	0.7656
5:g.88602535G > T	1	GG (695)	10226 ^A^±60.16	339.80 ± 2.72	3.34 ± 1.19	304.72^A^±2.18	2.98 ± 1.19
		GT (216)	10361 ^A^±75.72	335.97 ± 3.29	3.27 ± 1.74	306.41^A^±2.73	2.96 ± 1.74
		TT (11)	9416^B^±236.19	323.35 ± 9.59	3.47 ± 6.78	280.09^B^±8.42	2.99 ± 6.78
		*p*-value	<.0001**	0.081	0.9989	0.0065**	0.9999
	2	GG (474)	10642 ^A^±63.09	380.34^a^±2.84	3.59 ± 0.03	315.07^Aa^±2.07	2.97 ± 0.02
		GT (150)	11008^B^±86.68	389.2^b^±3.74	3.56 ± 0.04	323.41^Bb^±2.73	2.95 ± 0.02
		TT (4)	11107^AB^±394.01	396.3^ab^±15.94	3.57 ± 0.16	327.55^ab^±11.63	2.93 ± 0.09
		*p*-value	<.0001**	0.0129*	0.6534	0.0009**	0.4995
5:g.88630838T > C	1	CC (76)	10004 ^A^±102.56	331.94^a^±4.32	3.33 ± 2.64	297.94^a^±3.68	2.98 ± 2.64
		CT (386)	10168^B^±65.61	336.85^a^±2.91	3.33 ± 1.40	303.22^a^±2.37	2.99 ± 1.40
		TT (460)	10366^B^±65.01	342.44^b^±2.90	3.33 ± 1.33	307.76^b^±2.35	2.97 ± 1.33
		*p*-value	<.0001**	0.0057**	1	0.0339*	1
	2	CC (55)	10089 ^A^±120.89	366.73^Aa^±5.06	3.64 ± 0.05	299.87^A^±3.69	2.97 ± 0.03
		CT (265)	10624^B^±71.05	378.33^Ab^±3.14	3.58 ± 0.03	313.66^B^±2.28	2.96 ± 0.02
		TT (308)	10924 ^C^±70.14	388.76^Cc^±3.12	3.58 ± 0.03	323.05^C^±2.27	2.97 ± 0.02
		*p*-value	<.0001**	<.0001**	0.3979	<.0001**	0.9258
5:g.88630844C > A	1	AA (76)	10004 ^A^±102.56	331.94^a^±4.32	3.33 ± 2.64	297.94^a^±3.68	2.98 ± 2.64
		AC (386)	10168 ^A^±65.61	336.85^a^±2.91	3.33 ± 1.40	303.22^a^±2.37	2.99 ± 1.40
		CC (460)	10366^B^±65.01	342.44^b^±2.90	3.33 ± 1.33	307.76^b^±2.35	2.97 ± 1.33
		*p*-value	<.0001**	0.0057**	1	0.0339*	1
	2	AA (55)	10089 ^A^±120.89	366.73^Aa^±5.06	3.64 ± 0.05	299.87^A^±3.69	2.97 ± 0.03
		AC (265)	10624^B^±71.05	378.33^Ab^±3.14	3.58 ± 0.03	313.66^B^±2.28	2.96 ± 0.02
		CC (308)	10924 ^C^±70.14	388.76^Cc^±3.12	3.58 ± 0.03	323.05^C^±2.27	2.97 ± 0.02
		*p*-value	<.0001**	<.0001**	0.3979	<.0001**	0.9258
5:g.88630980T > C	1	CC (75)	10020 ^A^±103.15	331.94^a^±4.34	3.33 ± 2.66	298.32^a^±3.70	2.98 ± 2.66
		CT (385)	10167 ^A^±65.63	336.74^a^±2.91	3.33 ± 1.40	303.17^ab^±2.37	2.98 ± 1.40
		TT (462)	10361^B^±64.95	342.48^b^±2.90	3.33 ± 1.33	307.67^b^±2.35	2.97 ± 1.33
		*p*-value	<.0001**	0.0049**	1	0.0081**	1
	2	CC (54)	10038 ^A^±121.19	364.18^A^±5.07	3.63 ± 0.05	298.34^A^±3.70	2.97 ± 0.03
		CT (265)	10640^B^±71.07	379.16^B^±3.14	3.58 ± 0.03	314.07^B^±2.28	2.96 ± 0.02
		TT (309)	10918 ^C^±70.08	388.49^C^±3.11	3.58 ± 0.03	322.96^C^±2.27	2.97 ± 0.02
		*p*-value	<.0001**	<.0001**	1	<.0001**	0.8991

Note: The number in the table represents the mean ± standard deviation; the number in the bracket represents the number of cows for the corresponding genotype; *p*-value shows the significance for the genetic effects of SNPs; the superscript letters indicate the significance of different genotypes, involving the comparison between each two pairs; a, b, c within the same column with different superscripts means *p* < 0.05; and A, B, C within the same column with different superscripts means *p* < 0.01.

### Haplotype association analysis

**Figure 1.**
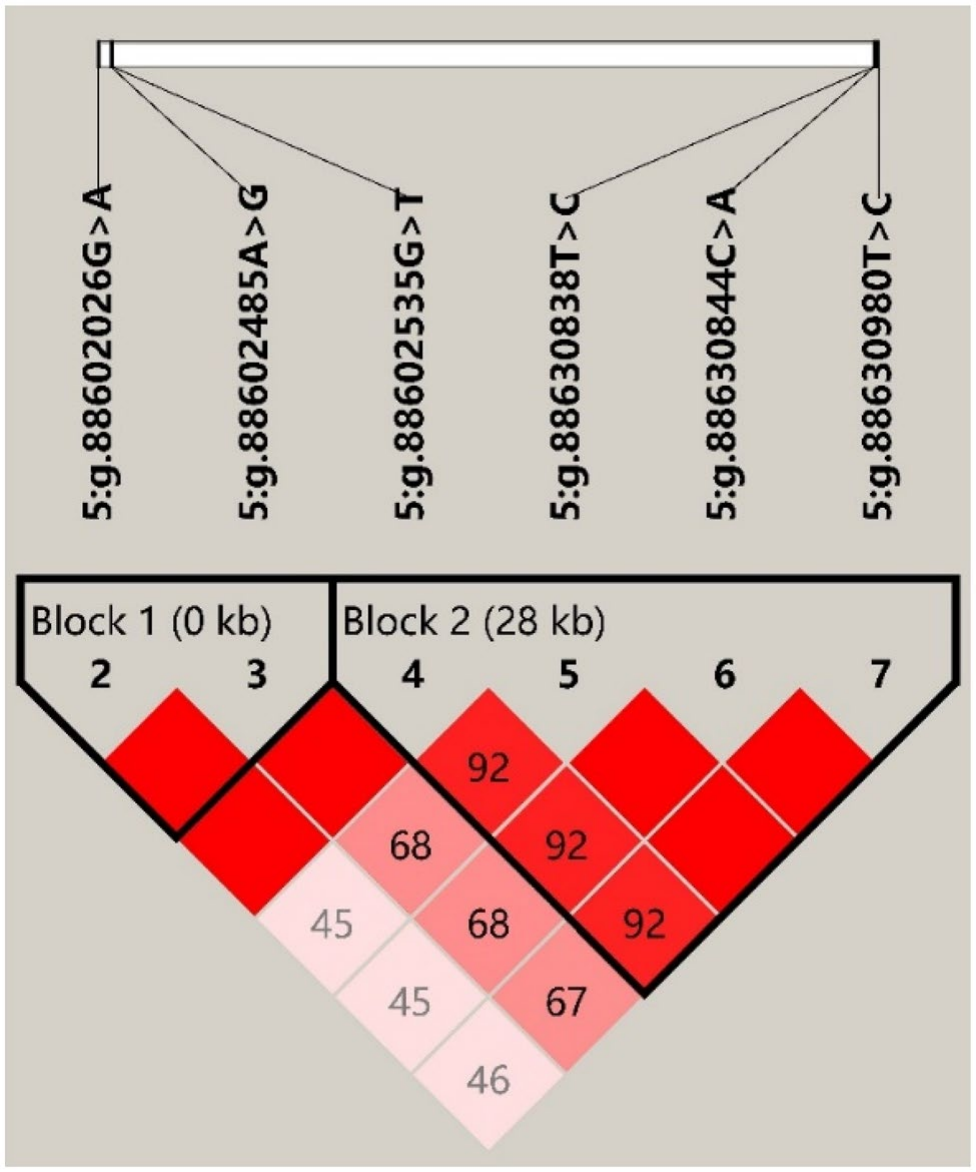
Linkage Disequilibrium estimated between SNPs in *GYS2* gene. The blocks indicate haplotype blocks and the text above the horizontal numbers is the SNP names. The values in the red boxes are pair-wise SNP correlations (D′), while bright red boxes without numbers indicate complete Linkage Disequilibrium (LD) (D′ = 1).

Through estimating the degree of LD among the seven identified SNPs by Haploview 4.2, six SNPs in *GYS2* were discovered to form two haplotype blocks (Fig. 1; Block 1: D′ = 1.00; Block 2: D′ = 0.92-1.00). In Block 1, three haplotypes were found, and the frequencies of H1 (GA), H2 (AA) and H3 (AG) were 0.554, 0.381 and 0.065, respectively. Block 2 consisted of five haplotypes, H1 (GCAC), H2 (GTCT), H3 (TTCT), H4 (TCAC) and H5 (GCAT), with frequencies of 0.287, 0.582, 0.126, 0.03 and 0.02, respectively. Moreover, the haplotype combinations had significant associations with the 305-day milk yield, fat yield, fat percentage, or protein yield in the two lactations (*p-*value ≤ 0.0349; Table 3). In the first lactation, Block 1 demonstrated significant associations with 305-day milk yield, fat and protein yield (*p-*value ≤ 0.005), and Block 2 had significant associations with 305-day milk yield, fat yield and fat percentage (*p-*value ≤ 0.0349). In the second lactation, both blocks were significantly associated with the 305-day milk yield, fat and protein yield, and fat percentage (*p-*value ≤ 0.0286).

**Table 3.** Haplotype analyses for *GYS2* gene.

Block	Lactation	Haplotype Combination	Milk Yield (kg)	Fat Yield (kg)	Fat Percentage (%)	Protein Yield (kg)	Protein Percentage (%)
Block1	1	H1H1 (276)	10144^Aa^±69.75	334.91^Aa^±3.07	3.33 ± 0.03	301.91^a^±2.24	2.99 ± 0.02
		H1H2 (399)	10383^Bb^±64.40	340.69^ab^±2.86	3.31 ± 0.03	306.6^ab^±2.08	2.96 ± 0.02
		H1H3 (71)	10141^ab^±104.93	332.34^Aa^±4.40	3.31 ± 0.04	299.85^ab^±3.21	2.97 ± 0.03
		H2H2 (128)	10402^Ab^±85.83	348.32^Bb^±3.66	3.37 ± 0.03	308.91^b^±2.67	2.98 ± 0.02
		H2H3 (47)	10250^ab^±121.70	345.56^ab^±5.06	3.38 ± 0.05	305.47^ab^±3.69	2.99 ± 0.03
		*p*-value	0.0002**	0.0001**	0.1465	0.005**	0.5163
	2	H1H1 (176)	10017^Aa^±100.84	332.52^Aa^±4.24	3.33^ab^±0.04	298.86^Aa^±3.09	2.99 ± 0.03
		H1H2 (272)	10306^ABb^±67.72	342.4^ABac^±2.99	3.34^ABa^±0.03	307.27^ACb^±2.18	2.99 ± 0.02
		H1H3 (54)	10180^ABab^±100.72	335.97^Aa^±4.23	3.32^ab^±0.04	305.12^ACab^±3.08	3.00 ± 0.03
		H2H2 (94)	10427^BCb^±67.59	349.57^Bb^±2.99	3.37^Aa^±0.03	311.25^BCbc^±2.18	2.99 ± 0.02
		H2H3 (31)	10648^Cc^±82.09	346.14^ABbc^±3.52	3.25^Bb^±0.03	314.12^Cc^±2.57	2.95 ± 0.02
		*p*-value	<.0001**	<.0001**	0.0053**	<.0001**	0.096
Block2	1	H1H1 (75)	10665^ab^±81.49	385.66 ± 3.55	3.64^a^±0.03	317.90 ± 2.59	2.99 ± 0.02
		H1H2 (306)	10812^a^±69.50	385.32 ± 3.08	3.58^ab^±0.03	319.99 ± 2.24	2.97 ± 0.02
		H1H3 (76)	10459^b^±120.43	373.56 ± 5.03	3.59^ab^±0.05	312.12 ± 3.66	3.00 ± 0.03
		H2H2 (311)	10734^ab^±97.89	381.99 ± 4.15	3.56^ab^±0.04	318.78 ± 3.03	2.98 ± 0.03
		H2H3 (140)	10773^ab^±148.40	371.10 ± 6.12	3.45^b^±0.06	314.99 ± 4.46	2.93 ± 0.04
		*p*-value	0.0349*	0.0227*	0.0333*	0.2136	0.4246
	2	H1H1 (54)	10030^Aa^±119.95	367.47^Aa^±5.02	3.67 ± 0.05	298.15^Aa^±3.66	2.98 ± 0.03
		H1H2 (215)	10624^Bb^±75.34	380.28^ACac^±3.31	3.58 ± 0.03	315.03^Bb^±2.41	2.97 ± 0.02
		H1H3 (49)	10691^Bbc^±125.26	390.62^BCbc^±5.22	3.67 ± 0.05	317.3^BCbc^±3.80	2.98 ± 0.03
		H2H2 (203)	10844^Bc^±74.99	389.33^BCb^±3.28	3.60 ± 0.03	323.26^Cc^±2.39	2.99 ± 0.02
		H2H3 (101)	11287^Dd^±96.50	397.72^Bb^±4.11	3.53 ± 0.04	334.38^Dd^±3.00	2.96 ± 0.03
		*p*-value	<.0001**	<.0001**	0.0286*	<.0001**	0.8146

Note: The number in the table represents the mean ± standard deviation; H means haplotype; in Block1, H1: GA, H2: AA, H3: AG; in Block2, H1: GCAC, H2: GTCT, H3: TTCT; the number in the bracket represents the number of cows for the haplotype combination; *p*-value shows the significance for the genetic effects of haplotype combination; the superscript letters indicate the significance of different genotypes, involving the comparison between each two pairs; a, b, c, d within the same column with different superscripts means *p* < 0.05; and A, B, C, D within the same column with different superscripts means *p* < 0.01.

### Prediction results of functional changes caused by SNPs

#### Regulation of the 5′ region SNPs on transcriptional activity

The TFBS changes of the two SNPs, 5:g.88602007G > A and 5:g.88602026G > A, located in the 5′ region of the *GYS2* gene, respectively, were predicted, using the Jaspar and Meme-suite software to obtain the common result (Table 4). Mutation from the allele G to A of 5:g.88602007G > A caused the appearance of the binding site (BS) for transcription factor (TF) myocyte enhancer factor 2 A (MEF2A) and myocyte enhancer factor 2D (MEF2D). For 5:g.88602026G > A, allele A created the BS for POU Class 2 Homeobox 2 (POU2F2) and eliminated the BS for NK3 Homeobox 1 (Nkx3-1).

**Table 4.** Transcription factor binding sites (TFBSs) prediction for SNPs in *GYS2* gene.

SNPs	Allele	Transcription factor	Relative score (≥ 0.80; Jasper)	*P*-value (≤ 0.01; Meme-suite)	Predicted Binding Site Sequence
5:g.88602007G > A	G				
	A	MEF2A	0.89	0.0030	TTCTATATATAACCT
		MEF2D	0.90	0.0044	TCTATATATAAC
5:g.88602026G > A	G	Nkx3-1	0.81	0.0008	ATACTTT
	A	POU2F2	0.85	0.0005	ATGATCTGCATAC

#### Alteration of missense mutations on protein structure and function

**Figure 2.**
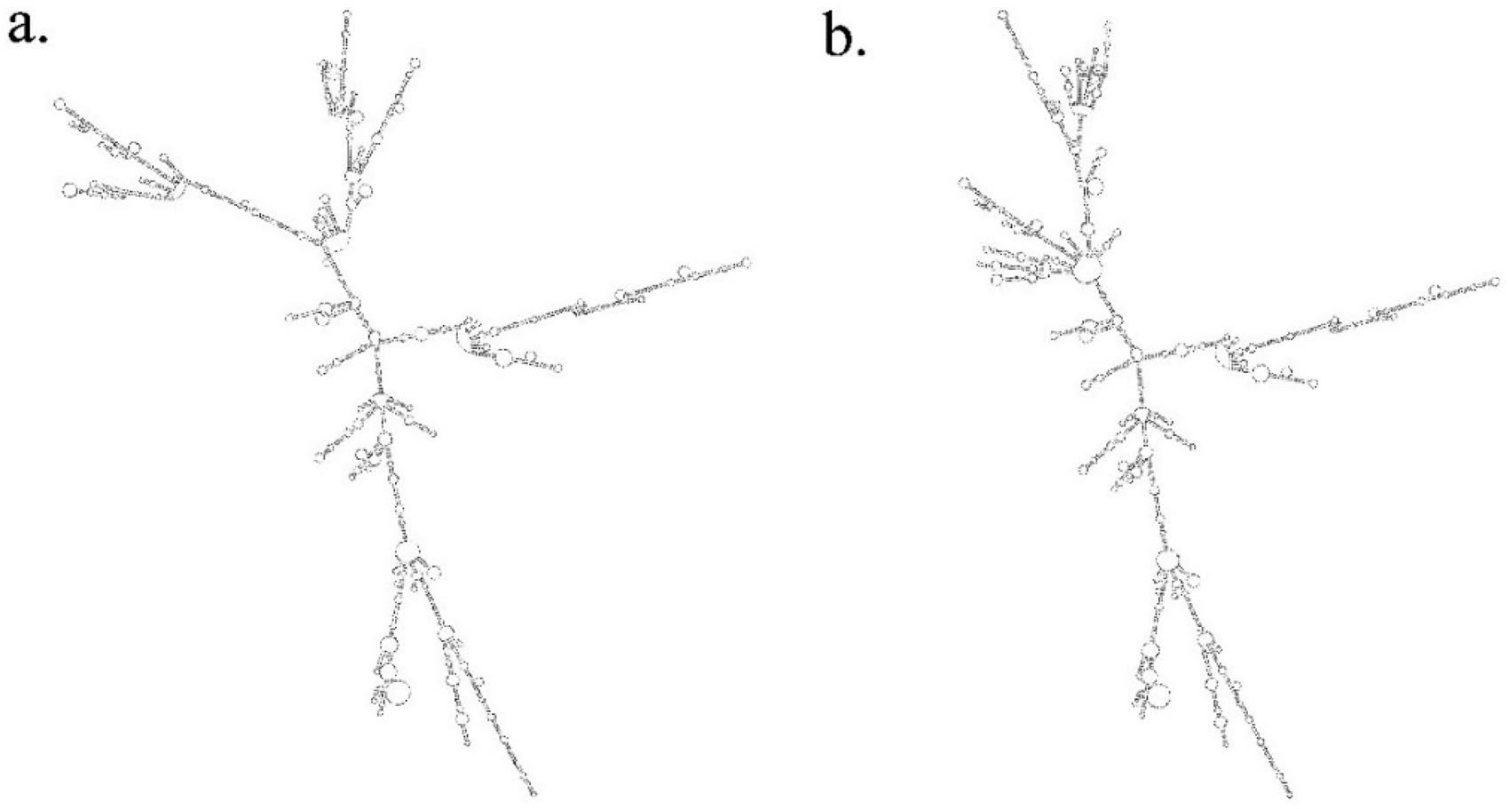
Secondary structure of minimum free energy of the predicted mRNA before and after mutation at the site 5:g.88602535G > T. (a) allele G of 5:g.88602535G > T; (b) allele T of 5:g.88602535G > T.

**Figure 3.**
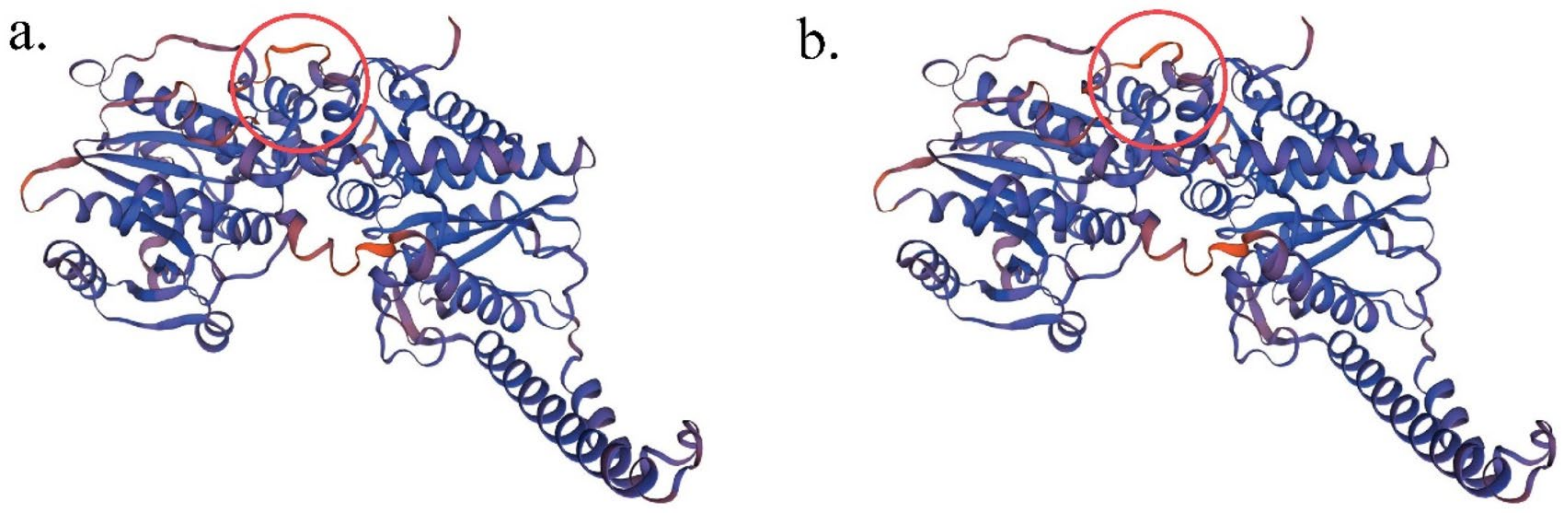
Tertiary structure of the predicted protein before and after mutation at the site 5:g.88602535G > T. (a) allele G of 5:g.88602535G > T; (b) allele T of 5:g.88602535G > T. The difference between the two structures is indicated by red circle.

There were three SNPs identified in the coding region of *GYS2* gene, one of which was a missense mutation, namely 5: g.88602535G > T. RNAfold prediction result revealed that as the allele altered from G to T, the secondary structure of mRNA transcribed was markedly changed (Fig. 2) and minimum free energy (MFE) of the structure changed from −805.10 kcal/mol to −807.00 kcal/mol. Besides, after the mutation of this site from G to T, the 17th amino acid in the translated amino acid sequence mutated from R to L. The protein secondary structure predicted by Prabi software changed subsequently, with the content of alpha helix, extended strand, beta turn and random coil different from 47.38% to 48.09%, 11.88% to 11.32%, 5.94% to 5.52% and 34.79% to 35.08%, respectively (Table 5). After modeling the protein tertiary structure by SWISS MODEL software, global quality scores in Cβ, all atom, solvation and torsion changed from −0.15 to −0.57, 1.15 to 1.09, 1.09 to 1.07, and −1.31 to −1.27, respectively (Table 5). A small change in 3D structure model could be observed (Fig. 3). Furthermore, ΔΔG related to protein stability was 0.15 kcal/mol through u-Pro prediction results. PolyPhen-2 and SMART software indicated that the mutation at this site was benign and did not fall into the functional domain of the corresponding protein, causing no changes in protein function. To conclude, the missense mutation changed the secondary structure of mRNA and the secondary and tertiary structure of protein, and affected protein stability as well.

**Table 5.** Predicted results of the changes of protein secondary structure, tertiary structure and function caused by the missense mutation.

SNP	Allele	Amino acid	Changes of protein secondary structure				Changes of protein Tertiary structure					Changes of protein stability	Changes of protein function
			Alpha helix (Hh)	Extended strand (Ee)	Beta turn (Tt)	Random coil (Cc)	GMQE	Cβ	All Atom	solvation	torsion	Mu-Pro (ΔΔG)	PolyPhen-2
5:g.88602535 G > T	G	Arg	47.38%	11.88%	5.94%	34.79%	0.82	−0.15	1.15	1.09	−1.31	0.15	Benign
	T	Leu	48.09%	11.32%	5.52%	35.08%	0.82	−0.57	1.09	1.07	−1.27		

## Discussion

### Valuable SNPs for milk production traits revealed by single marker and haplotype association analysis

Compared with single marker association analysis, haplotype analysis simplifies the complexity of genetic variation to a certain extent, and haplotype tends to be linked transmission, which is more effective than single marker transmission.^17^^,^^18^ Therefore, it has advantages in identifying and detecting variant haplotypes for complex traits.^19^ As the linked transmission of haplotypes tends to amplify the signal of association, in this study, we found two haplotype blocks composed of six SNPs were significantly associated with milk yield, fat yield, fat percentage, and protein percentage. Nevertheless, the consistency that both single marker and haplotype blocks have significant association with milk yield and fat yield reinforced the robustness of our findings and the reliability of the candidate gene *GYS2*’s significant genetic effects on milk production traits. The seven significant SNPs discovered in this study could provide effective genetic information for molecular breeding, such as serving as loci for gene editing, increasing their weight in genome selection, and being considered for inclusion in commercial chips, and have value for further functional validation.

### Mutations in the 5′ flanking regions affect milk production traits by affecting gene transcription

The transcription factor can target specific TFBS to regulate the transcript levels of its downstream genes, yet SNPs located at TFBSs may affect the binding of transcription factors, resulting in differences in gene expression among individuals with different genotypes.^20^ In this study, the changes of TFBSs were caused by the SNPs in the 5′ flanking regions of *GYS2* gene. For 5:g.88602007G > A, the BS of TF MEF2A and MEF2D appeared when the allele G mutated to A. The protein encoded by MEF2A is a DNA-binding transcription factor that activates many muscle-specific, growth factor-induced, and stress-induced genes.^21^ MEF2D is a transcriptional activator which found in numerous muscle-specific, growth factor- and stress-induced genes.^22^ Based on the fact that the fat percentage of cows with genotype GG were significantly higher than those with genotype AA in the second lactation, it was speculated that the AA type may affect the expression of *GYS2* gene by binding to the TF MEF2A and MEF2D, resulting in a decrease in milk fat percentage. For 5:g.88602026G > A, allele A created the BS for POU2F2 and eliminated the BS for Nkx3*-*1. POU2F2 regulates transcription in a number of tissues in addition to activating immunoglobulin gene expression, like regulating glycolytic reprogramming via PDPK1-dependent activation of PI3K/AKT/mTOR pathway.^23^ The transcription factor encoded by Nks-1 functions as a negative regulator of epithelial cell growth in prostate tissue.^24^ As the 305-day milk yield of AA genotype individuals were significantly higher than those of GG individuals in the first lactation, it was conjectured that the AA type might promote gene expression by binding to TF POU2F2, contributing to increases in 305-day milk yield. These findings suggest that the mutant site 5:g.88602007G > A and 5:g.88602026G > A could modulate the expression of *GYS2* to affect the milk production traits in dairy cattle by binding the changing TF.

### Missense mutations in the coding region affect milk production traits by affecting gene translation

5:g.88602535G > T located in the gene coding region was a missense mutation that altered the amino acid sequence which could affect proteins, the key medium for the transmission of information from the genome to the phenotype.^25^ Polymorphic alleles can markedly affect mRNA secondary structure and the caused alteration can modulate protein translation, and may also affect protein structure by affecting the binding and stability of chemical bonds.^26^^,^^27^ Some studies have linked human phenotypes to genotypic variation at the nucleotide level and changes in 3D protein structure.^28^ In this study, the prediction results showed that the mutation at 5: g. 88602535 G > T site caused changes in the secondary structure of mRNA as well as the secondary and tertiary structures of proteins, which might affect phenotype by changing protein activity. Meanwhile, association analysis results showed that 5:g.88602535G > T was significantly associated with milk and protein yields in the first lactation, and milk, fat and protein yields in the second lactation. It was speculated that this SNP may be a key site affecting the expression of *GYS2* gene leading to changes in milk production traits.

## Conclusion

In this study, seven SNPs were found in *GYS2* and significantly associated with milk production traits in dairy cows. Three SNPs among them, 5:g.88630838T > C, 5:g.88630844C > A and 5:g.88630980T > C were comparatively effective as having significant association with milk, fat and protein yields both in the first and second lactations. Notably, 5:g.88602007G > A and 5:g.88602026G > A, might regulate the expression of *GYS2* through changing TFBSs, and 5:g.88602535G > T might affect milk production traits by altering the structure and stability of the corresponding protein. These three SNP sites were proposed to be key causative mutations of *GYS2* affecting milk production traits, but their biological function mechanism needed further validation.

## Supplementary Material

Supplemental Material

Supplemental Material

## References

[1] Taylor JF, Taylor KH, Decker JE. Holsteins are the genomic selection poster cows. *Proc Natl Acad Sci U S A*. 2016;113(28):7690–7692. 27357662 10.1073/pnas.1608144113 PMC4948318

[2] Kadri NK, Guldbrandtsen B, Lund MS, et al. Genetic dissection of milk yield traits and mastitis resistance quantitative trait loci on chromosome 20 in dairy cattle. *J Dairy Sci*. 2015;98(12):9015–9025. 26409972 10.3168/jds.2015-9599

[3] Huang CW, Lin YT, Ding ST, et al. Efficient SNP discovery by combining microarray and lab-on-a-chip data for animal breeding and selection. *Microarrays (Basel)*. 2015;4(4):570–595. 27600241 10.3390/microarrays4040570 PMC4996412

[4] Bobbo T, Tiezzi F, Penasa M, et al. Short communication: Association analysis of diacylglycerol acyltransferase (DGAT1) mutation on chromosome 14 for milk yield and composition traits, somatic cell score, and coagulation properties in Holstein bulls. *J Dairy Sci*. 2018;101(9):8087–8091. 30007808 10.3168/jds.2018-14533

[5] Cobanoglu O, Kul E, Gurcan EK, et al. Determination of the association of GHR/AluI gene polymorphisms with milk yield traits in Holstein and Jersey cattle raised in Turkey. *Arch Anim Breed*. 2021;64(2):417–424. 34611546 10.5194/aab-64-417-2021 PMC8485837

[6] Ye W, Xu L, Li Y, et al. Single nucleotide polymorphisms of ALDH18A1 and MAT2A genes and their genetic associations with milk production traits of chinese holstein cows. *Genes (Basel)*. 2022;13(8):1437. 36011348 10.3390/genes13081437 PMC9407996

[7] Xu L, Shi L, Liu L, et al. Analysis of liver proteome and identification of critical proteins affecting milk fat, protein, and lactose metabolism in dariy cattle with iTRAQ. *Proteomics*. 2019;19(12):e1800387. 30903674 10.1002/pmic.201800387

[8] Roach PJ, Depaoli-Roach AA, Hurley TD, et al. Glycogen and its metabolism: some new developments and old themes. *Biochem J*. 2012;441(3):763–787. 22248338 10.1042/BJ20111416 PMC4945249

[9] Wathes DC, Cheng Z, Salavati M, et al. Relationships between metabolic profiles and gene expression in liver and leukocytes of dairy cows in early lactation. *J Dairy Sci*. 2021;104(3):3596–3616. 33455774 10.3168/jds.2020-19165

[10] Duplessis M, Blais L, Poisson W, et al. Technical note: extrapolation of hepatic glycogen concentration of the whole organ by performing a liver biopsy. *J Dairy Sci*. 2020;103(5):4858–4862. 32113751 10.3168/jds.2019-17905

[11] Nayeri S, Sargolzaei M, Abo-Ismail MK, et al. Genome-wide association for milk production and female fertility traits in Canadian dairy Holstein cattle. *BMC Genet*. 2016;17(1):75. 27287773 10.1186/s12863-016-0386-1 PMC4901445

[12] Kolbehdari D, Wang Z, Grant JR, et al. A whole genome scan to map QTL for milk production traits and somatic cell score in Canadian Holstein bulls. *J Anim Breed Genet*. 2009;126(3):216–227. 19646150 10.1111/j.1439-0388.2008.00793.x

[13] Boichard D, Grohs C, Bourgeois F, et al. Detection of genes influencing economic traits in three French dairy cattle breeds. *Genet Sel Evol*. 2003;35(1):77–101. 12605852 10.1186/1297-9686-35-1-77 PMC2732691

[14] Jiang L, Liu X, Yang J, et al. Targeted resequencing of GWAS loci reveals novel genetic variants for milk production traits. *BMC Genomics*. 2014;15(1):1105. 25510969 10.1186/1471-2164-15-1105 PMC4377845

[15] Frischknecht M, Pausch H, Bapst B, et al. Highly ­accurate sequence imputation enables precise QTL mapping in Brown Swiss cattle. *BMC Genomics*. 2017;18(1):999. 29284405 10.1186/s12864-017-4390-2 PMC5747239

[16] Khatib H, Zaitoun I, Wiebelhaus-Finger J, et al. The association of bovine PPARGC1A and OPN genes with milk composition in two independent Holstein cattle populations. *J Dairy Sci*. 2007;90(6):2966–2970. 17517737 10.3168/jds.2006-812

[17] Stram DO. Multi-SNP haplotype analysis methods for association analysis. *Methods Mol Biol*. 2017;1666:485–504. 28980261 10.1007/978-1-4939-7274-6_24

[18] Vadva Z, Larsen CE, Propp BE, et al. A new pedigree-based SNP haplotype method for genomic polymorphism and genetic studies. *Cells*. 2019;8(8):835. 31387299 10.3390/cells8080835 PMC6721696

[19] Abed A, Belzile F. Comparing single-SNP, Multi-SNP, and haplotype-based approaches in association studies for major traits in barley. *Plant Genome*. 2019;12(3):1–14. 10.3835/plantgenome2019.05.0036 PMC12810143 33016584

[20] Yu C-P, Li W-H. Predicting transcription factor binding sites and their cognate transcription factors using gene expression data. *Methods Mol Biol*. 2017;1629:271–282. 28623591 10.1007/978-1-4939-7125-1_17

[21] Xiao Q, Gan Y, Li Y, et al. MEF2A transcriptionally upregulates the expression of ZEB2 and CTNNB1 in colorectal cancer to promote tumor progression. *Oncogene*. 2021;40(19):3364–3377. 33863999 10.1038/s41388-021-01774-w PMC8116210

[22] Zhu B, Ramachandran B, Gulick T. Alternative pre-mRNA splicing governs expression of a conserved acidic transactivation domain in myocyte enhancer factor 2 factors of striated muscle and brain. *J Biol Chem*. 2005;280(31):28749–28760. 15834131 10.1074/jbc.M502491200

[23] Yang R, Wang M, Zhang G, et al. POU2F2 regulates glycolytic reprogramming and glioblastoma progression via PDPK1-dependent activation of PI3K/AKT/mTOR pathway. *Cell Death Dis*. 2021;12(5):433. 33931589 10.1038/s41419-021-03719-3 PMC8087798

[24] Papachristodoulou A, Rodriguez-Calero A, Panja S, et al. NKX3.1 localization to mitochondria suppresses prostate cancer initiation. *Cancer Discov*. 2021;11(9):2316–2333. 33893149 10.1158/2159-8290.CD-20-1765 PMC7611624

[25] Nussinov R, Tsai C-J, Jang H. Protein ensembles link genotype to phenotype. *PLoS Comput Biol*. 2019;15(6):e1006648. 31220071 10.1371/journal.pcbi.1006648 PMC6586255

[26] Duan J, Wainwright MS, Comeron JM, et al. Synonymous mutations in the human dopamine receptor D2 (DRD2) affect mRNA stability and synthesis of the receptor. *Hum Mol Genet*. 2003;12(3):205–216. 12554675 10.1093/hmg/ddg055

[27] Nackley AG, Shabalina SA, Tchivileva IE, et al. Human catechol-O-methyltransferase haplotypes modulate protein expression by altering mRNA secondary structure. *Science*. 2006;314(5807):1930–1933. 17185601 10.1126/science.1131262

[28] Botstein D, Risch N. Discovering genotypes underlying human phenotypes: past successes for mendelian disease, future approaches for complex disease. *Nat Genet*. 2003;33 Suppl(S3):228–237. 12610532 10.1038/ng1090

