# Supplementary figures and images for: Single nucleotide polymorphisms of GYS2 gene and its association with milk production traits of dairy cows
Source: Anim Biotechnol. 2024 Dec 4;35(1):2432966. doi: 10.1080/10495398.2024.2432966 (PMC12674338; doi:10.1080/10495398.2024.2432966)

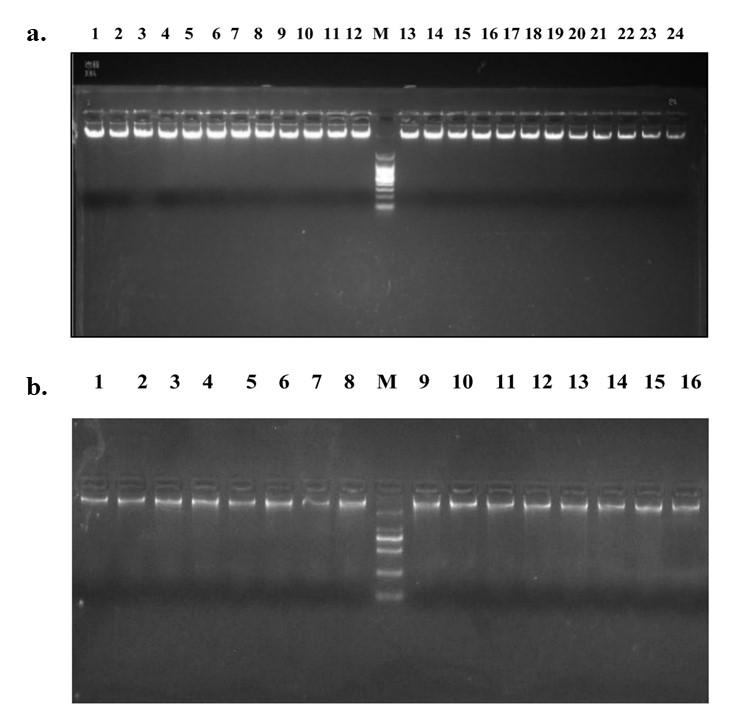

Supplement: Supplemental Material [file LABT_A_2432966_SM1683.jpg]
